# Activation of AMP-Activated Protein Kinase by Adenine Alleviates TNF-Alpha-Induced Inflammation in Human Umbilical Vein Endothelial Cells

**DOI:** 10.1371/journal.pone.0142283

**Published:** 2015-11-06

**Authors:** Yi-Fang Cheng, Guang-Huar Young, Jiun-Tsai Lin, Hyun-Hwa Jang, Chin-Chen Chen, Jing-Yi Nong, Po-Ku Chen, Cheng-Yi Kuo, Shao-Hsuan Kao, Yao-Jen Liang, Han-Min Chen

**Affiliations:** 1 Energenesis Biomedical Co., Ltd., New Taipei City, Taiwan; 2 Department of Life Science, Institute of Applied Science and Engineering, Catholic Fu-Jen University, New Taipei City, Taiwan; 3 Department of Internal Medicine, Collage of Medicine, National Taiwan University, Taipei, Taiwan; 4 Institute of Biochemistry and Biotechnology, Chung Shan Medical University, Taichung, Taiwan; Université catholique de Louvain, BELGIUM

## Abstract

The AMP-activated protein kinase (AMPK) signaling system plays a key role in cellular stress by repressing the inflammatory responses induced by the nuclear factor-kappa B (NF-κB) system. Previous studies suggest that the anti-inflammatory role of AMPK involves activation by adenine, but the mechanism that allows adenine to produce these effects has not yet been elucidated. In human umbilical vein endothelial cells (HUVECs), adenine was observed to induce the phosphorylation of AMPK in both a time- and dose-dependent manner as well as its downstream target acetyl Co-A carboxylase (ACC). Adenine also attenuated NF-κB targeting of gene expression in a dose-dependent manner and decreased monocyte adhesion to HUVECs following tumor necrosis factor (TNF-α) treatment. The short hairpin RNA (shRNA) against AMPK α1 in HUVECs attenuated the adenine-induced inhibition of NF-κB activation in response to TNF-α, thereby suggesting that the anti-inflammatory role of adenine is mediated by AMPK. Following the knockdown of adenosyl phosphoribosyl transferase (APRT) in HUVECs, adenine supplementation failed to induce the phosphorylation of AMPK and ACC. Similarly, the expression of a shRNA against APRT nullified the anti-inflammatory effects of adenine in HUVECs. These results suggested that the role of adenine as an AMPK activator is related to catabolism by APRT, which increases the cellular AMP levels to activate AMPK.

## Introduction

Inflammation-induced endothelial dysfunction is closely associated with many vascular diseases [1]. Vascular inflammation causes a wide range of diseases, including atherosclerosis, hypertension, ischemia/reperfusion injury, diabetes, myocardial infarction and chronic kidney disease [2–5]. It is initiated by the inflammatory activation of endothelial cells that express adhesion molecules and facilitate the tethering and rolling of leukocytes on the endothelial lining [6–8]. Activation of nuclear factor-kappa B (NF-κB) plays a central role in the inflammatory reactions of endothelial cells by up-regulating the expression of adhesion molecules and other inflammatory mediators [9]. Thus, the inhibition of NF-κB blocks endothelial inflammation by suppressing the expression of NF-κB- dependent genes and subsequent monocyte adhesion.

AMP-activated protein kinase (AMPK), a key sensor of energy homeostasis in eukaryotes, is mainly regulated by cellular AMP, which causes a conformational change that leads to phosphorylation by upstream kinases [10–12]. In addition to the regulation of energy homeostasis, recent studies have reported that AMPK is involved in modulating cellular stresses such as inflammatory response [13–15]. Activated AMPK can repress the NF-κB translocation as well as reduce the expression levels of NF-κB target genes and monocyte adhesion to endothelial cells [16]. Numerous studies also supported the anti-inflammatory function of AMPK activators in response to proinflammatory stimuli, such as a glucagon-like peptide-1 analog, 5-aminoimidazole-4-carboxamide ribonucleoside (AICAR), metformin, resveratrol and berberine [17–19].

Adenine phosphoribosyltransferase (APRT) is an important enzyme in the salvage pathway, which functions as a catalyst in the reaction between adenine and phosphoribosyl pyrophosphate (PRPP) to produce AMP. Our previous findings suggest that adenine represents a novel AMPK activator, which could ameliorate LPS-induced inflammation in microglial BV2 cells and enhance glucose uptake in NIH-3T3 cells via the activation of AMPK [20, 21]. However, the precise mechanism that underlies adenine-induced AMPK activation is still unknown.

In this study, we found that adenine reduces tumor necrosis factor (TNF)-α-stimulated monocyte adhesion by inhibiting NF-κB translocation in human umbilical vein endothelial cells (HUVECs). The administration of adenine induces the phosphorylation of AMPK and acetyl Co-A carboxylase (ACC); however, this is not so after the knockdown of APRT in HUVECs. Consistent with previous studies of TNF-α-stimulated monocyte adhesion, we found that the anti-inflammatory effect of adenine was dependent on AMPK and APRT. These results demonstrate that adenine-induced AMPK phosphorylation is mediated by APRT.

## Materials and Methods

### Reagents and chemicals

All reagents were purchased from Sigma-Aldrich (St. Louis, MO, USA) except where otherwise specified. Dulbecco's modified Eagle's medium (DMEM) and fetal bovine serum (FBS) were purchased from Invitrogen (Carlsbad, CA, USA). Adenine with a proprietary name of ENERGI-F704 was generously provided by Energenesis-Biomedical Co., Ltd. (New Taipei City, Taiwan).

### Cell culture

Human umbilical vein endothelial cell line (HUVEC, BCRC, H-UV001) was purchased from Food Industry Research and Development Institute, Hsin Chu, Taiwan. Cells were cultured in Dulbecco's modified Eagle's medium (DMEM) containing 10% fetal bovine serum (FBS), 4 mM L-glutamine, 2 mM sodium pyruvate and 0.01% penicillin/streptomycin (Invitrogen GibcoBRL, Carlsbad, CA, USA) at 37°C under 5% CO_2_. The cells used in this experiment were between passage 3 and 8.

### Cell viability assay

Cell viability was analyzed using 2,3-bis-(2-methoxy-4-nitro-5-sulfophenyl)- 2H-tetrazolium-5-carboxanilide (XTT) assay. Briefly, HUVECs were seeded in 96-well plates at 4× 10^5^ cells/mL for 18 h and subsequently treated with either adenine or AICAR at designated concentrations (0, 200, 600, 1200, 2400 and 4800 μM). After 24 h incubation, 50 μL of XTT reagent was added. The plates were then incubated for 4 h at 37°C in the dark. The absorbance was measured at 490 nm with a reference wavelength set at 690 nm using VersaMax ELISA microplate reader (Molecular device, Sunnyvale, CA, USA). Data was presented as relative absorbance values to untreated cells.

### Western blot assays

HUVECs were lysed using cell lysis buffer [10 mM Tris-HCl pH7.5, 150 mM NaCl, 1 mM ethylenediaminetetraacetic acid (EDTA), 0.5% (v/v) Triton-X 100, 1× Protease inhibitor cocktail (Roche, Basel, Switzerland), 1× PhosSTOP phosphatase inhibitor cocktail (Roche, Basel, Switzerland)] for 30 min at 4°C, and then centrifuged at 15,000×g for 1 min. The equal amount of cell lysates were resolved by sodium dodecyl sulfate polyacrylamide gel (SDS-PAGE) and then transfer to immobilon polyvinylidene difluoride (PVDF) membranes (Millipore, Bedford, MA, USA) as previous described [22]. The rabbit anti-phospho-AMPK (Thr172) antibody, rabbit anti-AMPK antibody, rabbit anti-COX-2 antibody, rabbit anti-ICAM-1 antibody and rabbit anti-VCAM-1 antibody were purchased from Cell signaling technology (Danvers, MA, USA). The rabbit anti-APRT antibody was purchased from Abcam (Cambridge, UK) and the mouse anti-β-actin antibody was purchased from Novus biologicals (Littleton, CO, USA). After the membranes were incubated with 1^st^ antibodies at 4°C overnight followed by the corresponding 2^nd^ antibody for 1 h at room temperature (RT), immunoreactive bands were detected by chemiluminescence (VisGlowTM, Visual Protein, Taipei, TW) and recorded using Kodak XAR-5 film (Rochester, NY, USA). The detected signals were scanned and then quantified using Image J software (http://image.nih.gov/ij/).

### Immunocytochemistry

HUVECs were seeded on glass coverslips in DMEM culture medium and treated with indicated concentration of adenine under TNF-α stimulation at 37°C for 6 h. Cells were fixed using 4% paraformaldehyde in PBS for 15 min and blocked with 3% BSA for 1 h at room temperature (RT). Then, cells were incubated with an anti-p65 antibody (1:200, #82452, Cell signaling technology) at 4°C overnight, followed by incubation with a goat anti-rabbit immunoglobulin G (IgG) conjugated to Alexa Fluor 488 (1:1000, ab150077, Abcam) for 2 h at RT. Coverslips were mounted with Ibidi mounting medium (Ibidi GmbH, Martinsried, Germany) and the pattern of immunostaining were analyzed using fluorescent microscopy (OLYMUS 1X71 research inverted system microscope).

### Enzyme-linked immunosorbent assay (ELISA)

Production of secreted IL-6 was measured using ELISA assay. The supernatant of cell culture was harvested and assessed. The cytokines IL-6 was evaluated using Mouse DuoSet ELISA kits (R&D Systems, Minneapolis, MN, USA). All the manipulations were performed following the manufacturer’s protocol.

### Monocyte-endothelial cell adhesion assay

The monocyte adhesion assay was performed as previously described with some modifications [23]. Briefly, HUVECs were seeded in 6-well plates at 4× 10^5^ cells/mL for 18 h and were stimulated with 10 μg/L of TNF-α for another 6 h in the present of adenine. For fluorescent labeling, THP-1 cells were incubated with 10 μM of Calcein AM (Sigma-aldrich) at 37°C for 30 min, and then were harvested by centrifugation (1,000×g, 5 min) and washed three times with PBS. The fluorescent THP-1 cells were suspended in medium and were added into TNF-α stimulated HUVECs with indicated treatments. After 30 min, non-adhering THP-1 cells were washed twice with PBS and THP-1 cells bound to HUVECs were imaged by OLYMUS 1X71 microscope.

### Construction of shAPRT and shAMPK expressing cell lines

HUVECs were cultured to 80% confluency and transfected with small hairpin RNA (shRNA) of APRT or AMPK using Turbofect (Thermo scientific, Waltham, USA) according to the manufacturer’s instructions. For knockdown the expression of APRT and AMPK, shRNA against human APRT 5’GCTGGTTGAGCAGCGGATCCGCAGCTTCC3’, AMPKα1 5’TCTGGTGTGGATTATTGTCACAGGCATAT3’ and non-targeting shRNA (shNT) were constructed in pGFP-V-RS vectors were purchased from Origene Technologies (Origene, MD, USA). The knockdown HUVECs were selected using 2 μg/mL puromycin (Sigma-aldrich) according to the manufacturer’s protocols, and the knockdown efficiency in HUVECs were confirmed by western blot analysis (S1 Fig and S2 Fig).

### Statistical analysis

Data were presented as means ± S.E.M. Comparison between treatments was performed using GraphPad PRISM software version 5.00 (San Diego, CA) using one-way ANOVA with Tukey post hoc test or two-way ANOVA with Bonferroni post hoc test. Statistical significance was accepted when *P*<0.05.

## Results

### Cytotoxic effect of adenine on HUVECs

**Fig 1:**
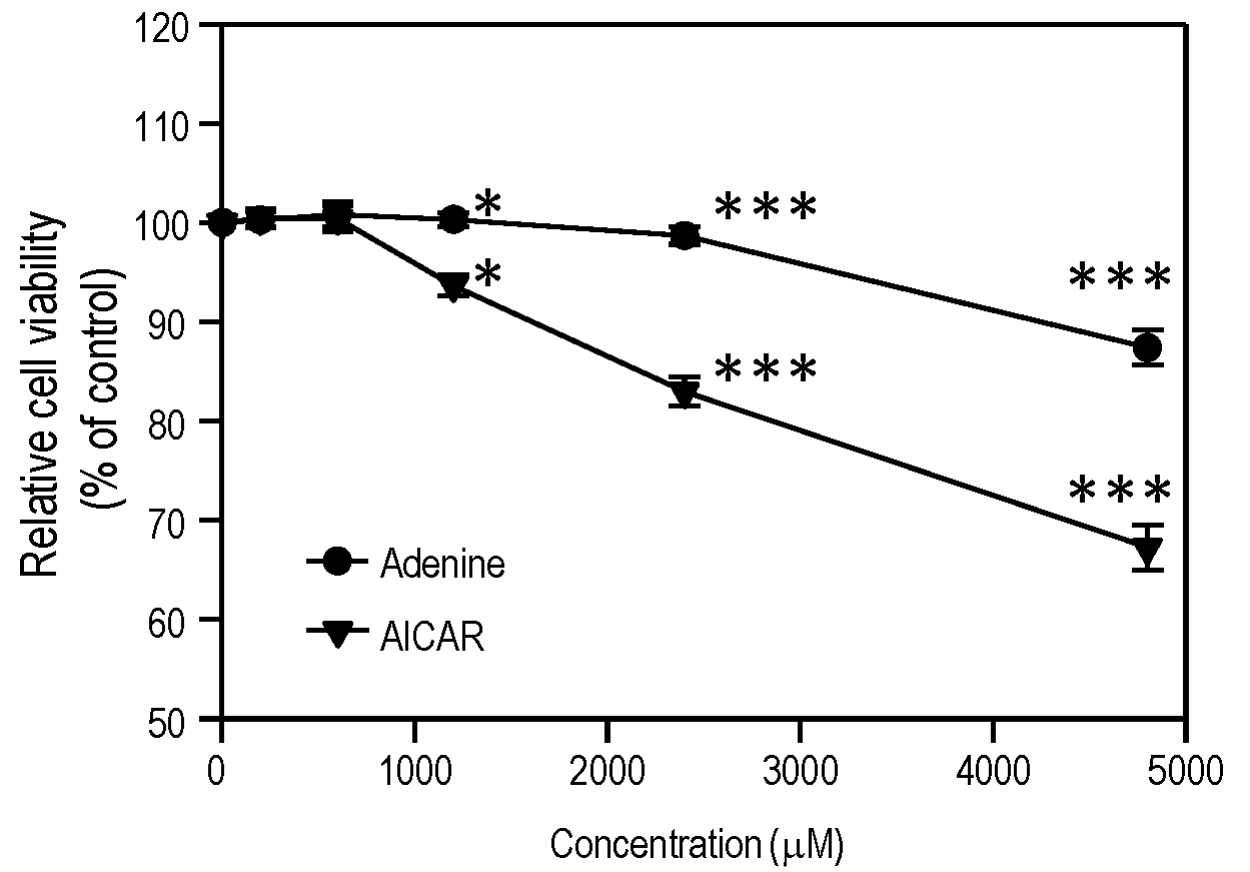
*In vitro* cytotoxicity of adenine and AICAR on HUVECs. Cells were treated by adenine or AICAR at different concentrations or solvent alone for 24 h. The cell viability of each condition was analyzed using XTT assay; Statistical significance was determined by two-way ANOVA followed by ad-hoc Bonferroni post hoc tests; all data are plotted as mean ± S.E.M (n = 3). *, *P*<0.05; ***, *P*<0.001.

We determined whether the *in vitro* cytotoxic effects of AMPK activators on HUVECs might be related to cell stress. The XTT cell viability assay was used to assess the *in vitro* cytotoxic effect of adenine in HUVECs after treatment for 24 h. As shown in Fig 1, adenine caused dose-dependent cytotoxicity at 4800 μM, whereas AICAR had cytotoxic effects at concentrations above 1200 μM.

### Time course- and dose- dependent effects of adenine on HUVECs

**Fig 2:**
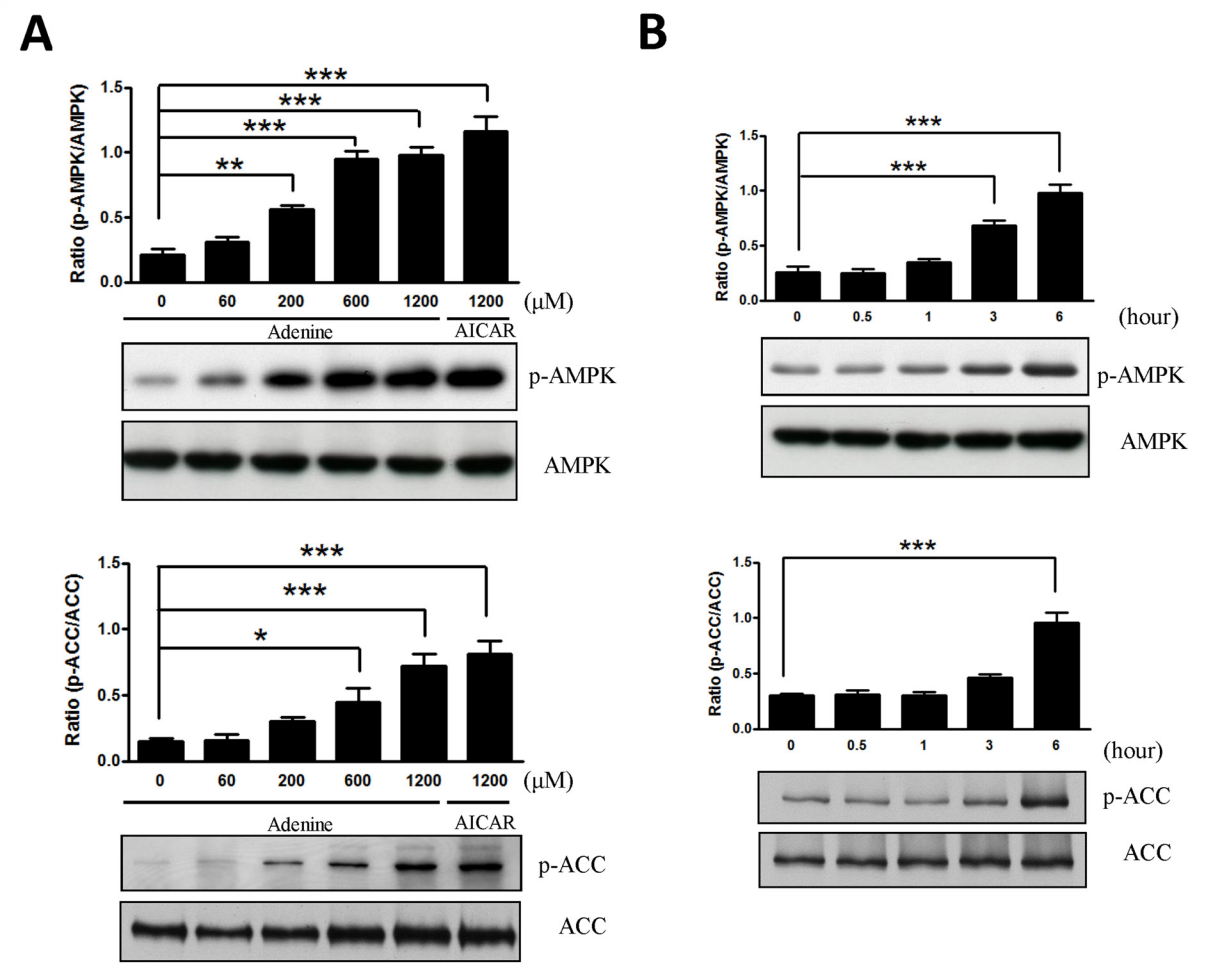
Adenine activated AMPK in HUVECs. (A) HUVEC cells were incubated for 6 h with various concentrations of adenine and compared with 1200 μM AICAR. (B) HUVEC cells were incubated with 600 μM adenine for the times indicated. Cell lysates were used to determine the phosphorylation of AMPK and ACC by western blot using antibodies specific for the phosphorylated protein. The level of total AMPK and ACC were also assessed as controls for loading. Statistical significance was determined by one-way ANOVA followed by Tukey post hoc tests; all data are plotted as mean ± S.E.M. (n = 5). *, *P*<0.05; **, *P*<0.01; ***, *P*<0.001.

To determine whether adenine activates AMPK, HUVECs were exposed to various concentrations of adenine and 1200 μM AICAR for 6 h before assessing AMPK phosphorylation at Thr172 using western blot analysis. As shown in Fig 2, the phosphorylation of both AMPK and its downstream target, ACC, were increased after adenine administration in a time- and dose-dependent manner.

### Adenine inhibited the NF-κB-dependent expression of IL-6, COX-2, ICAM-1 and VCAM-1 in TNF-α-activated HUVECs

**Fig 3:**
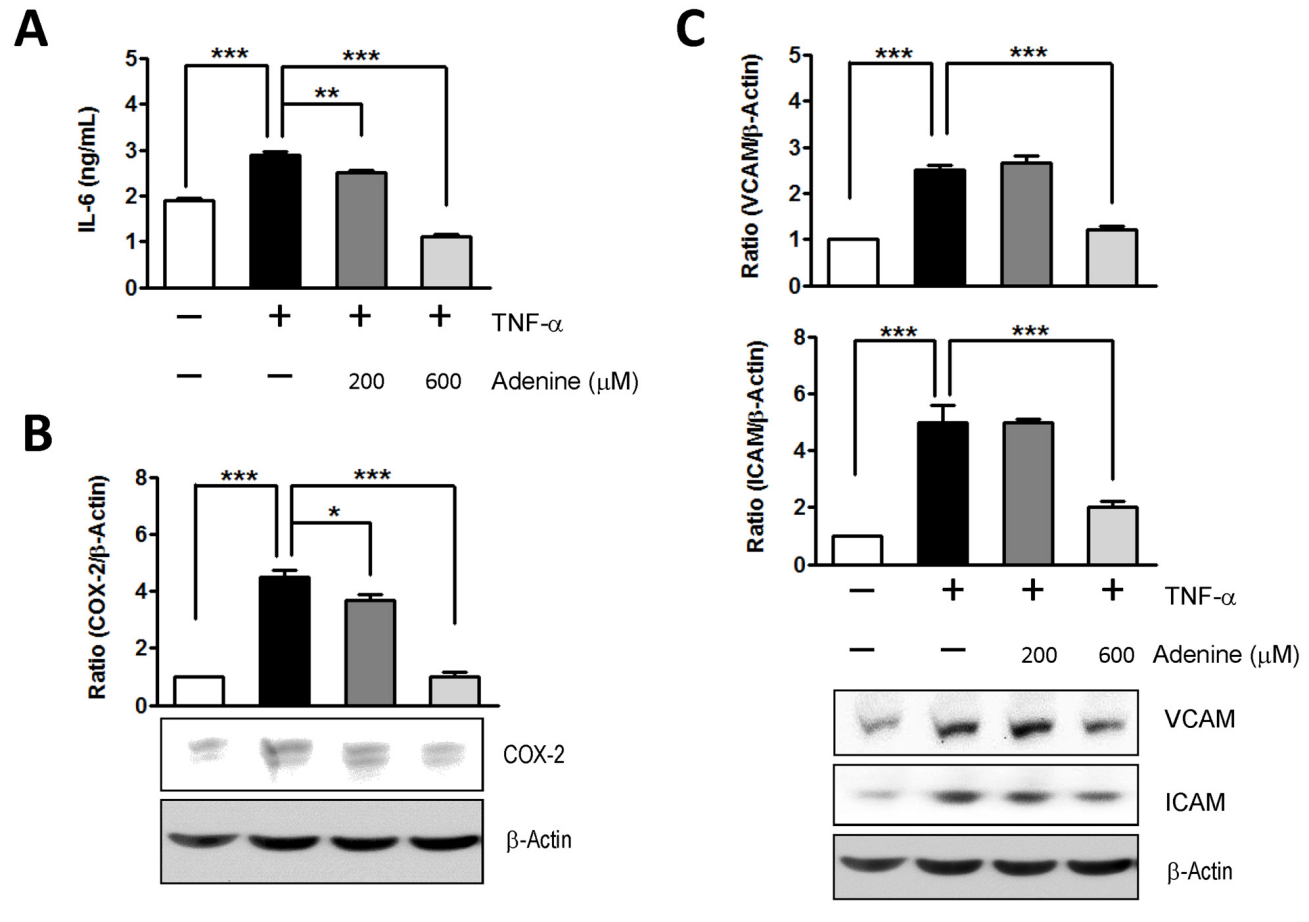
Effects of adenine on the NF-κB targeting gene expressions in response to TNF-α. HUVECs were treated with 10 μg/L of TNF-α in the presence or absence of adenine for 6 h. (A) The level of IL-6 in cultural media of each condition was analyzed using ELISA. Cell lysates collected from each condition were used to determine the expression of (B) COX-2, (C) VCAM and ICAM using western blot analysis. Statistical significance was determined by one-way ANOVA followed by Tukey post hoc tests; all data are plotted as mean ± S.E.M. (n = 3). *, *P*<0.05; **, *P*<0.01; ***, *P*<0.001.

It has been reported that the activation of AMPK reduces TNF-α-stimulated adhesion molecules by inhibiting the activity of NF-κB and via p65 translocation to the nucleus in endothelial cells [24]. Therefore, we investigated the effects of adenine on the expression of NF-κB target genes such as IL-6, COX-2, ICAM-1, and VCAM-1, which were induced by the NF-κB activator TNF-α in HUVECs. As shown in Fig 3A, adenine suppressed the secretion of IL-6 from TNF-α-activated HUVECs in a dose-dependent manner. Similarly, the administration of adenine resulted in a dramatic decrease in COX-2, ICAM-1 and VCAM-1 protein expression in response to TNF-α stimulation compared with the control group (Fig 3B and 3C).

### Adenine reduced the translocation of NF-κB after the stimulation of TNF-α in HUVECs

**Fig 4:**
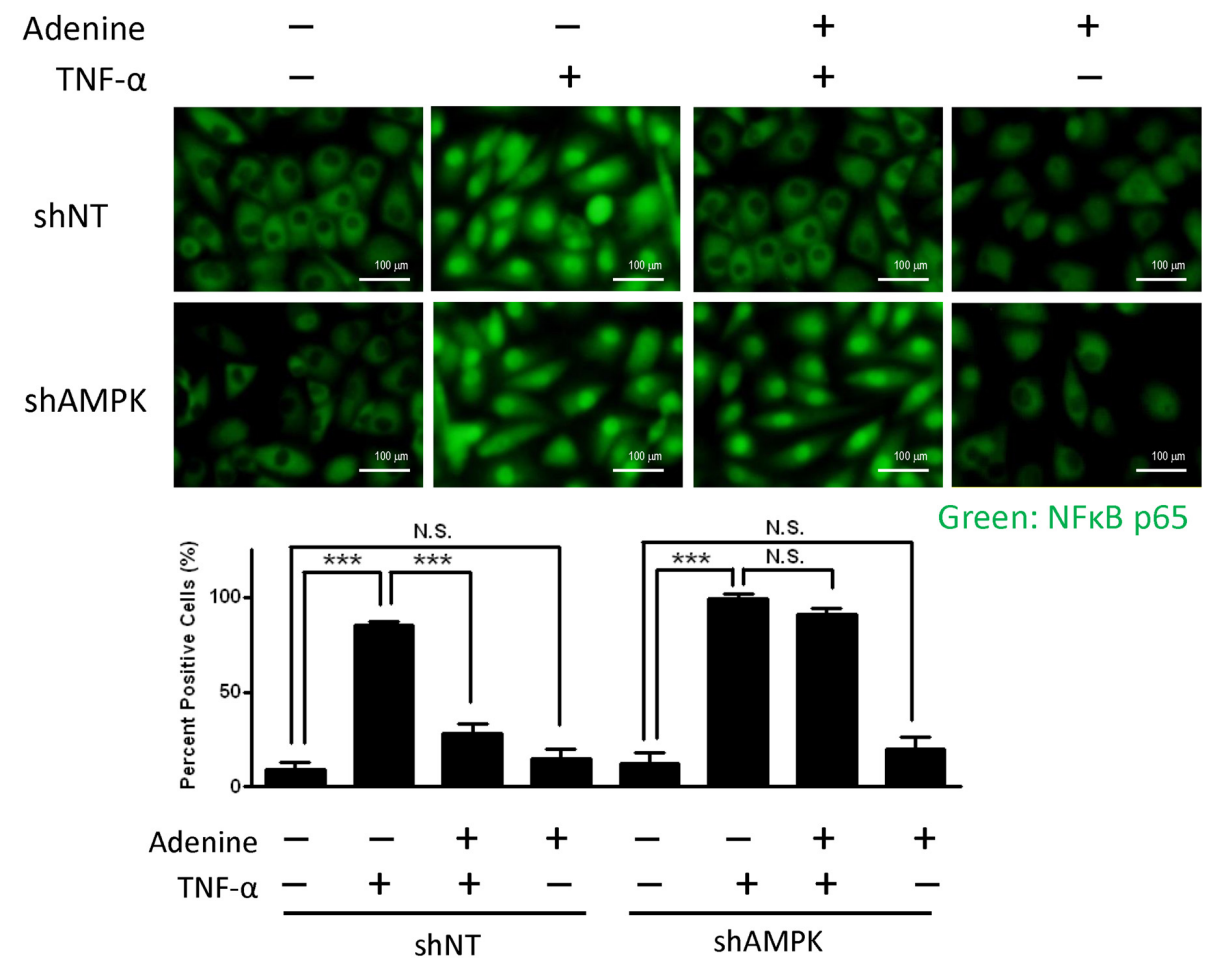
Effects of adenine on NF-κB translocation in response to TNF-α. shNT HUVECs or shAMPK HUVECs were treated with 10 μg/L of TNF-α in the presence or absence of 600 μM adenine for 6 h. Top: representative images depicting the location of NF-κB subunit p65 as described in “Material and methods”, Scale bar: 100 μM. Bottom: quantification of NF-κB subunit p65 translocation into nuclei of HUVECs. Statistical significance was determined by one-way ANOVA followed by Tukey post hoc tests; all data are plotted as mean ± S.E.M. (n = 5). ***, *P*<0.001; N.S., no significance.

The expression of adhesion molecules is NF-κB dependent; therefore, we also analyzed the mechanism that allows adenine to inhibit the expression of adhesion molecules by determining its effects on the translocation of NF-κB in TNF-α-stimulated HUVECs. As shown in Fig 4, the nuclear translocation of p65 in HUVECs was triggered by the stimulation of TNF-α, but this was reversed by treatment with adenine. In addition, the suppression of NF-κB translocation by adenine was demonstrated by the knockdown of the α1 subunit of AMPK. Genetic inhibition of AMPK failed to prevent the nuclear translocation of p65 following adenine treatment, thereby indicating that the suppressed expression of NF-κB targeting genes by adenine was mediated by AMPK.

### Adenine inhibited TNF-α-induced monocyte adhesion

**Fig 5:**
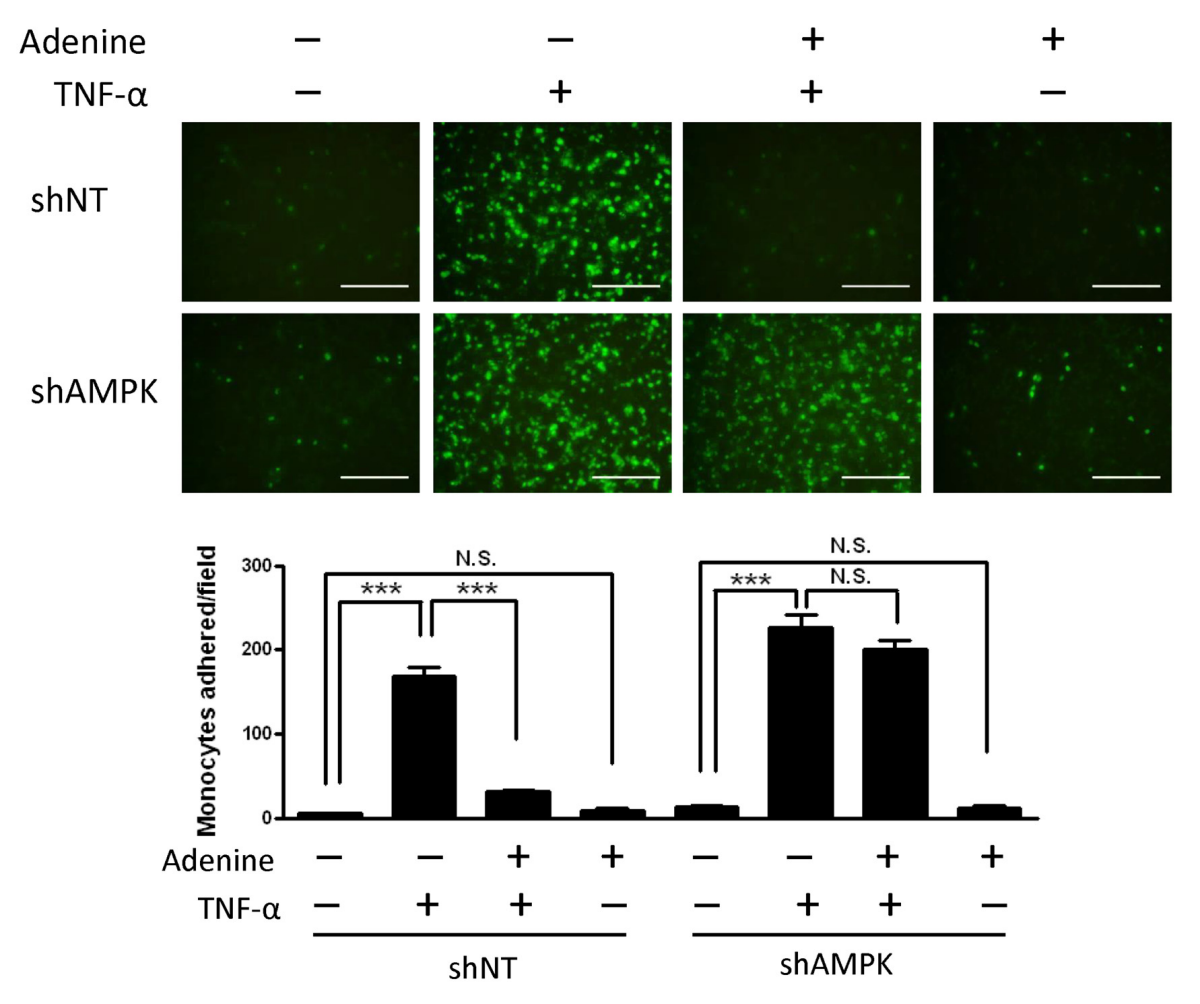
Effects of adenine on monocyte adhesion to HUVECs in response to TNF-α. shNT HUVECs or shAMPK HUVECs were treated with 10 μg/L of TNF-α for 6 h in the presence or absence of 600 μM adenine. Top: representative images depicting monocytes to the HUVEC cells as described in “Material and methods”, Scale bar: 200 μM. Bottom: quantification of monocyte adhesion to HUVEC cells. Statistical significance was determined by one-way ANOVA followed by Tukey post hoc tests; all data are plotted as mean ± S.E.M. (n = 5). ***, *P*<0.001; N.S., no significance.

To explore the functional impact of adenine on the interaction between endothelial cells and monocytes, we examined the adhesion of THP1 to TNF-α-stimulated HUVECs in the presence or absence of adenine for 6 h. In contrast to the control group, inhibition of monocyte adhesion occurred after adenine treatment in a dose-dependent manner (Fig 5). Treatment with 600 μM adenine caused a 70% decrease in monocyte adhesion relative to that in the control cells, whereas knockdown of the α1 subunit of AMPK had no effect. Overall, these results suggest that adenine inhibits the monocyte adhesion mediated by AMPK, thereby preventing the nuclear translocation of NF-κB in response to TNF-α.

### Adenine induced the phosphorylation of AMPK via ARPT

**Fig 6:**
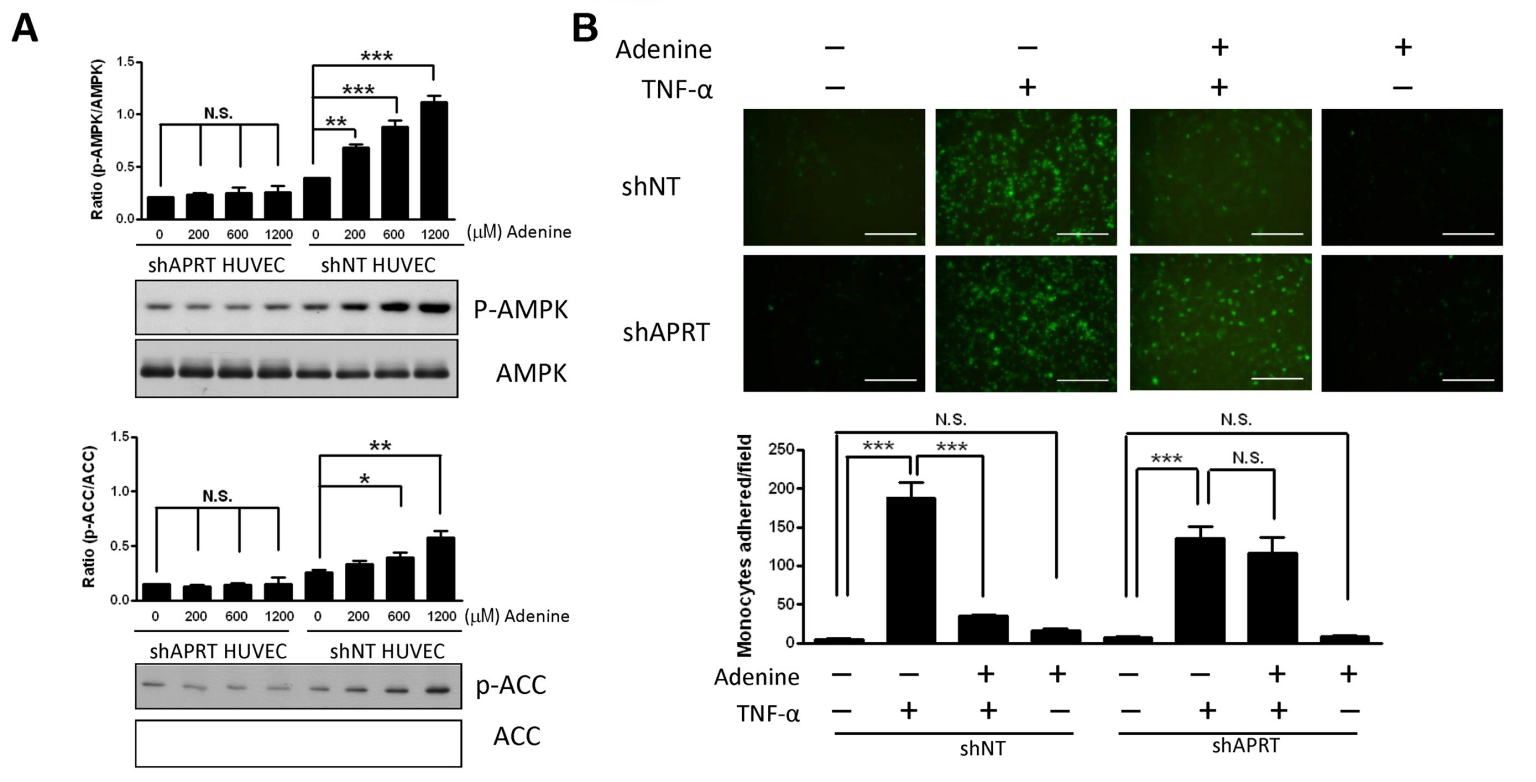
Effect of adenine induced AMPK activation and monocyte adhesion by APRT in HUVECs. (A) shNT HUVECs or shAPRT HUVECs were treated with various concentration of adenine for 6 h. Cell lysates collected from each condition were used to determine the phosphorylation of AMPK and ACC by western blot using antibodies specific for the phosphorylated protein. (B) shNT HUVECs or shAPRT HUVECs were exposed to 10 μg/L of TNF-α for 6 h in the presence or absence of 600 μM adenine. Top: representative images depicting monocytes to the HUVEC cells as described in “Material and methods”, Scale bar: 200 μM. Bottom: quantification of monocyte adhesion to HUVEC cells. Statistical significance was determined by one-way ANOVA followed by Tukey post hoc tests; all data are plotted as mean ± S.E.M. (n = 5). ***, *P*<0.001; N.S., no significance.

The utilization of adenine depends on the activity of APRT via the incorporation of PRPP into AMP [25]. To determine whether the effect of adenine-induced AMPK phosphorylation is mediated through the activity of APRT, we used retroviral short hairpin RNA (shRNA) against APRT in HUVECs. The expression of knockdown APRT was verified by western blot analysis and compared with the non-targeted shRNA (shNT) in HUVECs. We found that HUVECs transfected with a shAPRT plasmid exhibited a 92% reduction in APRT compared with the shNT HUVECs (S1B Fig). As shown in Fig 6A (Right panel), the levels of phosphorylated AMPK and its downstream target ACC increased in a dose-dependent manner following adenine treatment in shNT HUVECs, whereas they were unchanged in shAPRT HUVECs (Fig 6A, Left panel). This suggests that adenine-induced AMPK phosphorylation is mediated by the activity of APRT. Similarly, the effect of adenine on the adhesion between THP-1 cells and HUVECs was not changed by knockdown in HUVECs (Fig 6B). These results indicate that adenine mediates the TNF-α-induced adhesion ability between HUVECs and THP-1 cells via APRT.

## Discussion

The activation of endothelial AMPK signaling plays a critical role in the anti-inflammatory response induced by different stimuli. A decrease in AMPK activity, however, is associated with increased inflammation [16]. It has been reported that AMPK activators such as AICAR and metformin indirectly attenuate cytokine-induced NF-κB activation by suppressing the activity of IKK to stabilize the NF-κB-IκBα complex [24, 26]. In the present study, the anti-inflammatory effects of adenine are similar to those observed in our previous analysis of microglia following LPS treatment [20]. However, the molecular mechanism that underlies the effect of adenine on AMPK activation remains unknown. We suggest that this mechanism involves adenine supplementation, leading to an increase in cellular AMP levels, which in turn activates AMPK. To the best of our knowledge, the membrane-localized APRT is the only route that converts adenine into AMP via the incorporation of PRPP [25, 27, 28].

We investigated the toxicological effects of adenine in HUVECs. Adenine is a cell permeable compound and its uptake rate depends on the activity of APRT [27]. Excess of adenine (e.g. millimolar concentration) may be cytotoxic to cells by disrupting the cellular purine balance, thereby depleting the guanine pool and increasing the ratio of [AMP] relative to [GMP]. The administration of adenine significantly increases the phosphorylation of Thr172 in the α1-subunit in both a dose- and time-dependent manner (Fig 2). Our results demonstrate that adenine had a similar effect to AICAR, which induced the phosphorylation of AMPK and ACC (Fig 2). We also investigated whether the anti-inflammatory effects of adenine were related to an AMPK-independent pathway. After the knockdown of AMPK α1 in HUVECs, the administration of adenine failed to attenuate monocyte adhesion in inflamed HUVECs (Fig 5), thereby suggesting that the anti-inflammatory effects of adenine were mediated by AMPK.

In inflamed HUVECs, adenine down-regulated the production of proinflammatory cytokines and adhesion molecules by inhibiting the translocation of NF-κB [24, 26]. Activation of AMPK by adenine in TNF-α-treated HUVECs was found to be associated with preventing decreases in IκBα due to inhibition of the nuclear translocation of NF-κB (S3 Fig). Furthermore, the up-regulation of NF-κB downstream genes including IL-6, COX-2, ICAM-1 and VCAM-1 under TNF-α induction was reversed by adenine. These results suggest that adenine has the capacity to suppress TNF-α-induced NF-κB activation and AMPK activation by increasing the stability of the NF-κB-IκBα complex in HUVECs. In addition, it has been reported that AMPK activation can enhance the activity of SIRT1 by increasing intracellular NAD^+^ level [29]. For the p65 subunit of the NF-κB complex, deacetylation at Lysine310 facilitates methylation at Lysine314 and 315 to enhance the degradation of the p65 subunit by the ubiquitin-proteasome system [30, 31]. In previous studies, we also observed increases in the NAD^+^ level and decreases in the protein level of the p65 subunit of the NF-κB complex in inflammatory microglial cells after adenine treatment [20]. Overall, our results support the hypothesis that adenine can activate AMPK without cell type restrictions, and thus adenine may attenuate the translocation of NF-κB by simultaneously increasing the degradation of the p65 subunit and stabilizing IκB. Therefore, adenine may be capable of modulating the NF-κB proinflammatory signaling pathway, and it could have therapeutic applications against inflammatory diseases.

AMPK is known to play a vital role in the regulation of endothelial function as well as in metabolism modulation [32]. AMPK activation leads to increases in the activity of endothelial nitric oxide synthase [33–36], and it protects endothelial cells against metabolic and inflammatory stress. During inflammation in cardiovascular diseases, the endothelium encounters excessive amounts of chemokines or cytokines, which may lead to enhanced leukocyte infiltration and the upregulated expression of adhesion molecules [37, 38]. Significant expression of ICAM-1, VCAM-1 and E-selectin in endothelial cells is induced by various inflammatory stimuli including LPS and TNF-α, which are mediated by the NF-κB signaling pathway [39–41]. It has been reported that AMPK activators such as AICAR and metformin can reduce the surface levels of adhesion molecules in endothelial cells [26], which agrees with our finding that the upregulated adhesion molecules, including ICAM-1 and VCAM-1, in TNF-α-treated HUVECs were attenuated by adenine in a dose-dependent manner. We also observed that treatment with adenine decreased the number of adhesion numbers of THP-1 cells that interacted with TNF-α-treated endothelial cells. In addition, IL-6, a cytokine that is the hallmark of vascular inflammation, has been demonstrated to enhance the adhesion of monocytes with endothelial cells by inducing ICAM-1 expression via the STAT3 signaling cascade [42–45] and monocyte activation [3]. We suggest that the decreased secretion of IL-6 in TNF-α-activated HUVECs after adenine treatment contributes to the lower expression of adhesion molecules in the present study. Therefore, our results support previous investigations of the roles of AMPK in response to different stimuli [19].

The main finding of the present study is that adenine can activate AMPK in HUVECs via the conversion of APRT. We suggest that adenine-mediated AMPK activation occurs via the conversion of adenine into AMP, thereby inducing the phosphorylation of AMPK. Our results indicate that knockdown of APRT in HUVECs impaired the protective effects of adenine. Both AMPK and APRT are required for adenine to suppress the adhesion of THP-1 cells to inflamed HUVECs; therefore, the anti-inflammatory effects of adenine on HUVECs depend on AMPK via APRT.

In conclusion, we reveal that the administration of adenine increases the cellular AMP levels via catabolism by APRT, which in turn activates AMPK. Adenine, once known as Vitamin B4, is one of the simplest AMPK activators reported to date. For the first time, we demonstrate that the effect of adenine-mediated cell signaling on AMPK activation occurs via catabolism by APRT. Further *in vivo* studies should aim to target different disease models to identify suitable AMPK activators.

## Supporting Information

S1 Fig The knockdown efficiency of shAMPK in HUVECs. HUVECs infected with lentiviral non-targeting shRNA (shNT) or with different shRNAs specific for AMPKα1 (#1, #2, #3, #4), knockdown efficiency was determined by (A) western analysis in cell lysates. (B) Quantification of western blot of total AMPK protein level was normalized to β-Actin. Statistical significance was determined by one-way ANOVA followed by Tukey post hoc tests; all data are plotted as mean ± S.E.M. (n = 5). *, *P*<0.05; ***, *P*<0.001; N.S., no significance.
(TIF)

S2 Fig The knockdown efficiency of shAPRT in HUVECs. HUVECs infected with lentiviral non-targeting shRNA (shNT) or with different shRNAs specific for APRT (#1, #2, #3, #4), knockdown efficiency was determined by (A) western analysis in cell lysates. (B) Quantification of western blot of total APRT protein level was normalized to β-Actin. Statistical significance was determined by one-way ANOVA followed by Tukey post hoc tests; all data are plotted as mean ± S.E.M. (n = 5). *, *P*<0.05; ***, *P*<0.001; N.S., no significance.
(TIF)

S3 Fig Effects of adenine on protein expression of IκBα in TNF-α-activated HUVECs. Cells were incubated with 10 μg/L of TNF-α in the presence or absence of 600 μM adenine for 6 h. Cell lysates collected from each condition were used to determine the expression of IκBα using western blot analysis. Statistical significance was determined by one-way ANOVA followed by Tukey post hoc tests; all data are plotted as mean ± S.E.M. (n = 3). **, *P*<0.01; ***, *P*<0.001.
(TIF)
