# Supplementary figures and images for: Activation of AMP-Activated Protein Kinase by Adenine Alleviates TNF-Alpha-Induced Inflammation in Human Umbilical Vein Endothelial Cells
Source: PLoS One. 2015 Nov 6;10(11):e0142283. doi: 10.1371/journal.pone.0142283 (PMC4636334; doi:10.1371/journal.pone.0142283)

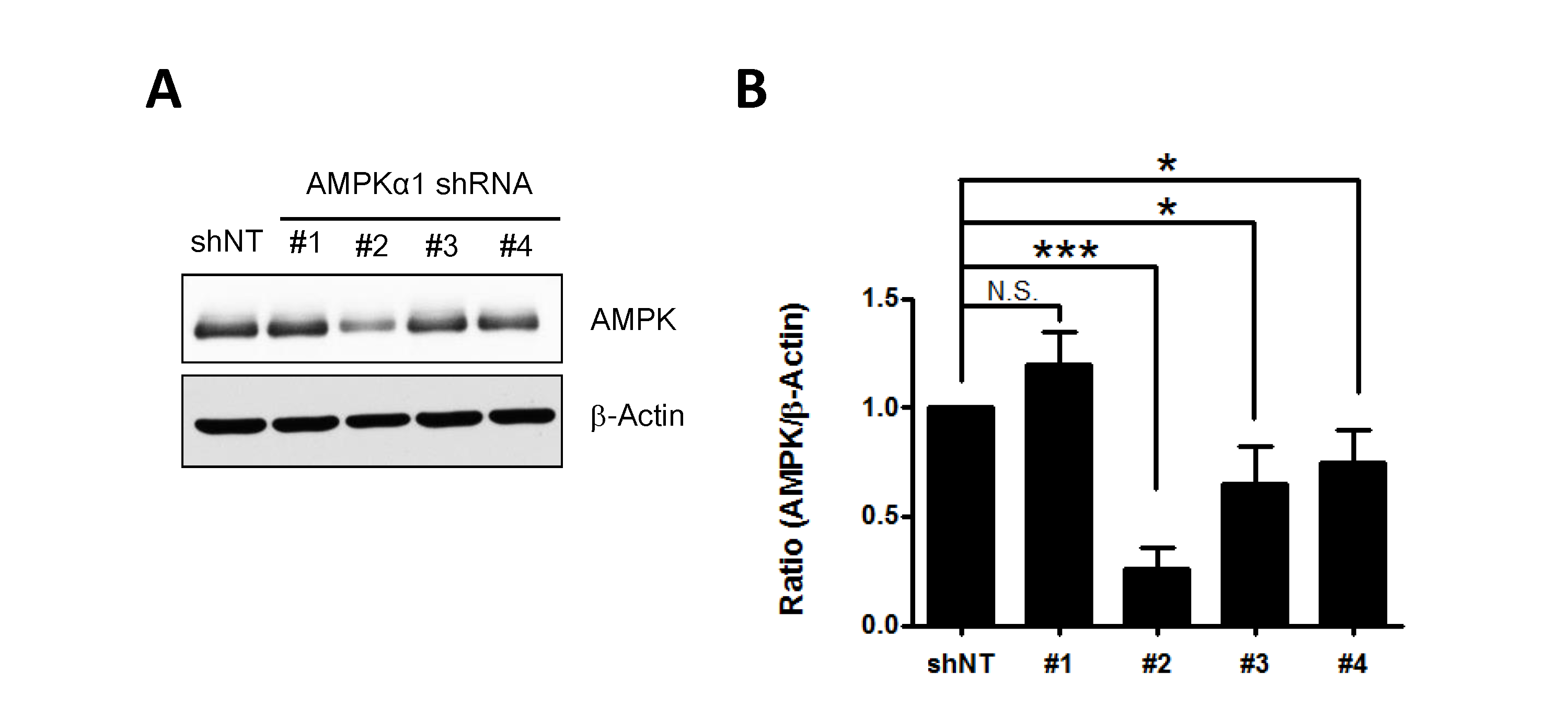

Supplement: S1 Fig — HUVECs infected with lentiviral non-targeting shRNA (shNT) or with different shRNAs specific for AMPKα1 (#1, #2, #3, #4), knockdown efficiency was determined by (A) western analysis in cell lysates. (B) Quantification of western blot of total AMPK protein level was normalized to β-Actin. Statistical significance was determined by one-way ANOVA followed by Tukey post hoc tests; all data are plotted as mean ± S.E.M. (n = 5). *, P<0.05; ***, P<0.001; N.S., no significance. (TIF) [file pone.0142283.s001.tif]

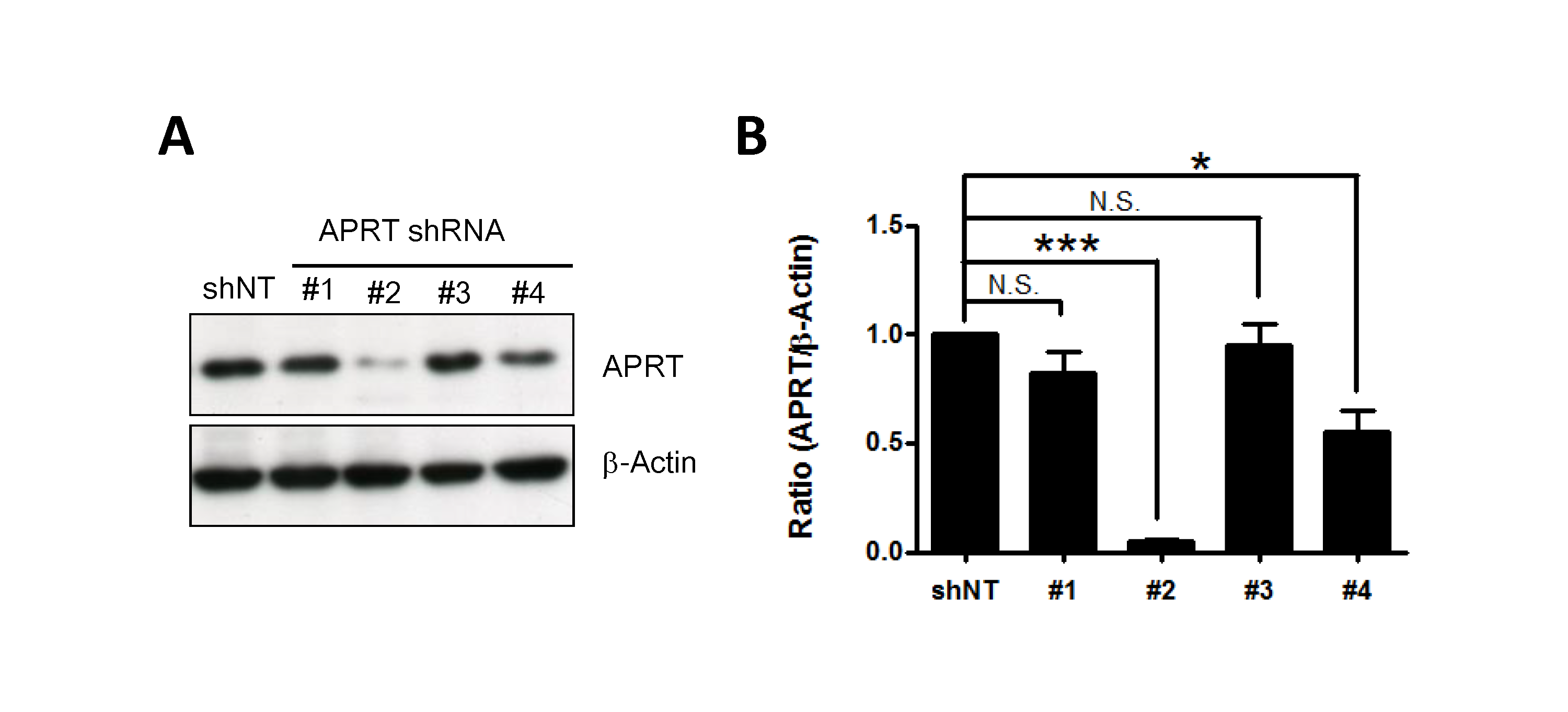

Supplement: S2 Fig — HUVECs infected with lentiviral non-targeting shRNA (shNT) or with different shRNAs specific for APRT (#1, #2, #3, #4), knockdown efficiency was determined by (A) western analysis in cell lysates. (B) Quantification of western blot of total APRT protein level was normalized to β-Actin. Statistical significance was determined by one-way ANOVA followed by Tukey post hoc tests; all data are plotted as mean ± S.E.M. (n = 5). *, P<0.05; ***, P<0.001; N.S., no significance. (TIF) [file pone.0142283.s002.tif]

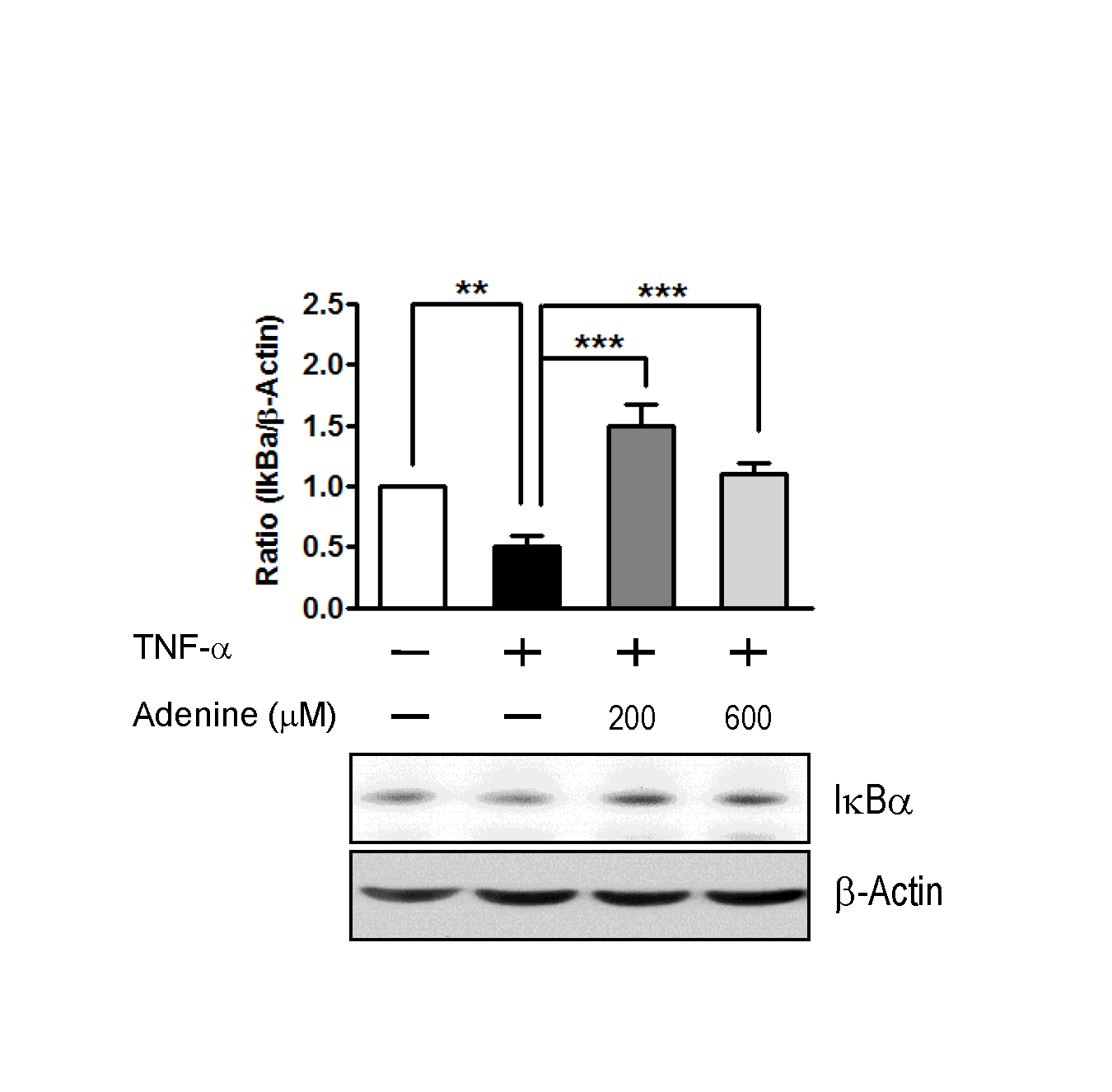

Supplement: S3 Fig — Cells were incubated with 10 μg/L of TNF-α in the presence or absence of 600 μM adenine for 6 h. Cell lysates collected from each condition were used to determine the expression of IκBα using western blot analysis. Statistical significance was determined by one-way ANOVA followed by Tukey post hoc tests; all data are plotted as mean ± S.E.M. (n = 3). **, P<0.01; ***, P<0.001. (TIF) [file pone.0142283.s003.tif]
